# AuNPs/CNC Nanocomposite with A “Dual Dispersion” Effect for LDI‐TOF MS Analysis of Intact Proteins in NSCLC Serum Exosomes

**DOI:** 10.1002/advs.202307360

**Published:** 2024-01-15

**Authors:** Liang Shan, Yongxia Qiao, Lifang Ma, Xiao Zhang, Changqiang Chen, Xin Xu, Dan Li, Shiyu Qiu, Xiangfei Xue, Yongchun Yu, Yinlong Guo, Kun Qian, Jiayi Wang

**Affiliations:** ^1^ Department of Clinical Laboratory Shanghai Chest Hospital Shanghai Jiao Tong University School of Medicine No. 241, West Huaihai Road Shanghai 200030 P. R. China; ^2^ School of Public Health Shanghai Jiao Tong University School of Medicine No. 227, South Chongqing Road Shanghai 200025 P. R. China; ^3^ Shanghai Institute of Thoracic Oncology Shanghai Chest Hospital Shanghai Jiao Tong University School of Medicine No. 241, West Huaihai Road Shanghai 200030 P. R. China; ^4^ National Center for Organic Mass Spectrometry in Shanghai Shanghai Institute of Organic Chemistry Chinese Academy of Sciences No. 345, Lingling Road Shanghai 200032 P. R. China; ^5^ State Key Laboratory for Oncogenes and Related Genes School of Biomedical Engineering Institute of Medical Robotics and Med‐X Research Institute Shanghai Jiao Tong University No. 1954, Huashan Road Shanghai 200030 P. R. China; ^6^ Faculty of Medical Laboratory Science College of Health Science and Technology Shanghai Jiao Tong University School of Medicine No. 227, South Chongqing Road Shanghai 200025 P. R. China

**Keywords:** AuNPs, biomarker, cellulose nanocrystal, intact protein analysis, LDI‐TOF MS, NSCLC

## Abstract

Detecting exosomal markers using laser desorption/ionization time‐of‐flight mass spectrometry (LDI‐TOF MS) is a novel approach for examining liquid biopsies of non‐small cell lung cancer (NSCLC) samples. However, LDI‐TOF MS is limited by low sensitivity and poor reproducibility when analyzing intact proteins directly. In this report, gold nanoparticles/cellulose nanocrystals (AuNPs/CNC) is introduced as the matrix for direct analysis of intact proteins in NSCLC serum exosomes. AuNPs/CNC with “dual dispersion” effects dispersed and stabilized AuNPs and improved ion inhibition effects caused by protein aggregation. These features increased the signal‐to‐noise ratio of [M+H]^+^ peaks by two orders of magnitude and lowered the detection limit of intact proteins to 0.01 mg mL^–1^. The coefficient of variation with or without AuNPs/CNC is measured as 10.2% and 32.5%, respectively. The excellent reproducibility yielded a linear relationship (*y* = 15.41*x* – 7.983, *R* ^2^ = 0.989) over the protein concentration range of 0.01 to 20 mg mL^–1^. Finally, AuNPs/CNC‐assisted LDI‐TOF MS provides clinically relevant fingerprint information of exosomal proteins in NSCLC serum, and characteristic proteins S100 calcium‐binding protein A10, Urokinase plasminogen activator surface receptor, Plasma protease C1 inhibitor, Tyrosine‐protein kinase Fgr and Mannose‐binding lectin associated serine protease 2 represented excellent predictive biomarkers of NSCLC risk.

## Graphical abstract

A strategy to reduce AuNP cluster and protein aggregation in the sample using AuNPs/CNC, which improved the signal sensitivity and reproducibility of LDI‐TOF MS analysis for intact proteins is introduced. Through AuNPs/CNC assisted LDI‐TOF MS analysis, exosome protein fingerprint can effectively distinguished between healthy individuals and NSCLC patients. Moreover, five exosomal proteins represented excellent predictive biomarkers of NSCLC risk.

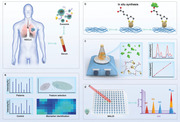

## 1 Introduction

Early diagnosis of non‐small cell lung cancer (NSCLC) relies on conventional biomarkers, low‐dose computed tomography, and biopsies.^[^ ^1^ ^]^ However, biomarkers and imaging are not sensitive enough in the diagnosis of NSCLC, and only 15% of new cases are diagnosed at an early stage.^[^ ^1^, ^2^ ^]^ Although squamous cell carcinoma antigen, neuron‐specific enolase, carcinoembryonic antigen, and cytokeratin 19 fragment are widely used as biomarkers for NSCLC diagnosis, false‐positive findings often occur because of benign lesions, infections, and pregnancy.^[^ ^3^ ^]^ For imaging, the sensitivity and specificity of computed tomography (CT) scanning for predicting mediastinal nodal metastases of NSCLC are approximately 55% and 81%, respectively, indicating the capacity of CT scanning is limited. Positron emission tomography scanning predicts mediastinal metastasis with 77% sensitivity and 86% specificity, however, the final diagnosis still requires biopsies.^[^ ^4^ ^]^ Although taking a biopsy is the “gold standard” for NSCLC diagnosis, this method is an invasive procedure, which may cause pain, severe infections, or dissemination of tumor cells. In recent years, there has been growing interest in using a liquid biopsy approach for early screening and diagnosis of NSCLC. Liquid biopsy is an in vitro diagnostic technique that uses body fluid as material to obtain tumor biology information, and the tested substances include circulating tumor cells (CTCs), circulating tumor DNA (ctDNA), and exosomes.^[^ ^5^, ^6^, ^7^ ^]^ Exosomes contain proteins, lipids, and nucleic acids, which are pivotal for communication between tumor cells and the microenvironment.^[^ ^8^, ^9^ ^]^ From a diagnosis standpoint, exosomes are far more abundant than CTCs, and the vesicular structure also protects active contents from degradation.^[^ ^8^ ^]^ Moreover, exosomal proteins provide direct information during NSCLC initiation and progression in “real‐time”, which has higher diagnostic sensitivity and specificity than ctDNA.^[^ ^9^ ^]^ However, present immunological assays are limited by the type of antibodies and complicated protein analysis procedures required. Thus, current studies of exosome markers for NSCLC mainly target non‐coding RNA, not proteins.

Compared with immunological assays, laser desorption/ionization time‐of‐flight mass spectrometry (LDI‐TOF MS) has the advantages of automation and lower cost, and the soft ionization technology used matrices make LDI‐TOF MS suitable for analyzing high‐mass molecules such as proteins.^[^ ^11^, ^12^, ^13^ ^]^ Currently, nanostructured materials are used in matrix‐free LDI‐MS for analyzing various biomolecules. These nanomaterials have high absorption coefficients in the ultraviolet (UV)‐vis range, which are ideal candidates for supporting LDI, such as gold nanoparticles (AuNPs), carbon nanomaterials, and porous silicon.^[^ ^14^, ^15^, ^16^, ^17^, ^18^, ^19^, ^20^, ^21^ ^]^ Nanomaterials are mainly used as matrices to analyze small molecules because these materials provide a source of ionization that ensures minimal interference in the low mass range.^[^ ^22^, ^23^, ^24^, ^25^, ^26^, ^27^, ^28^, ^29^ ^]^ Prompted by rapid progress in techniques, nanomaterials in different derivatized forms are also used as LDI matrices for analyzing peptides and proteins.^[^ ^30^ ^]^ For example, silica nanoparticles functionalized with cysteine, sulfobetaine, and amine alkoxysilanes have been used as the co‐matrix for matrix laser desorption/ionization time‐of‐flight mass spectrometry (MALDI‐TOF MS) analysis of proteins over the mass range of 2–60 kDa. A significant increase in the signal‐to‐noise ratio was observed when the colloidal stability of silica nanoparticles was achieved in a matrix solution.^[^ ^31^ ^]^ Moreover, functionalized fullerenes have the advantages of high ionization efficiency and small molar ratios of the matrix/analyte compared with organic acid matrices. However, because of the uneven dispersion of polar analytes in non‐polar fullerenes, the sensitivity of fullerenes for protein analysis is still lower than conventional organic acid matrices.^[^ ^31^ ^]^ Overcoming this issue by using ion mobility MS to pre‐separate carbon cluster ions from bioanalyte ions before TOF experiments is beneficial. An alternative is the deposition of fullerene MALDI samples by spraying.^[^ ^32^ ^]^ AuNPs can also act as a matrix in LDI‐TOF MS analysis of peptides and proteins because of their remarkable thermal conductivity and great optical adsorption efficiency.^[^ ^33^, ^34^, ^35^ ^]^ Huang et al. performed LDI‐MS analysis of thrombin at femtomolar concentrations using aptamer‐modified AuNPs as the matrix. After thrombin was selectively captured by the aptamer‐modified AuNPs, the complexes were deposited onto a nitrocellulose membrane to eliminate background noise. The binding of thrombin suppressed the ionization efficiency of the AuNPs, and thus, the decrease in the signal intensity of AuNP clusters represents a potential labeling indicator of thrombin. Although this approach enabled high‐sensitivity analysis of thrombin in low abundance, it did not directly analyze proteins and was prone to yielding false‐positive or false‐negative results.^[^ ^36^ ^]^ LDI is accompanied by the production of a particular amount of AuNP clusters, leading to the suppression of the signals of the analytes.^[^ ^37^ ^]^ Furthermore, AuNPs frequently form an inhomogeneous distribution on the target plate, leading to poor point‐to‐point reproducibility.^[^ ^38^ ^]^ The cellulose nanocrystal (CNC) is a nitrocellulose with a rod‐shaped structure and has attracted increasing attention because of its high aspect ratio, large specific surface area, richness in hydroxyl groups, and biocompatibility.^[^ ^36^, ^40^ ^]^ Recently, CNCs were used as a suitable stabilizer in the synthesis of metal nanoparticles in an aqueous medium.^[^ ^40^, ^41^, ^42^ ^]^ Moreover, carboxymethyl cellulose with a high degree of carboxymethyl substitution involves pH‐sensitive interactions with AuNPs and reduced aggregation.^[^ ^43^ ^]^ Herein, the dispersion of AuNPs in a CNC aqueous medium (AuNPs/CNC) was prepared by a one‐step method by reducing HAuCl_4_. We hypothesized that using homogeneous AuNPs/CNC nanoparticles should reduce interference from cluster ions and yield higher reproducibility.

**Figure 1:**
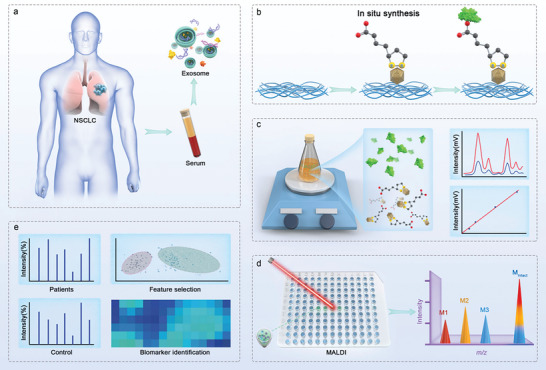
Graphical abstract. a) Exosome‐isolated patient serum with NSCLC. b) Synthesis of AuNPs/CNC and c) the process of sample preparation. d) LDL‐TOF MS analysis of intact proteins. e) Biomarker identification.

A further major challenge of protein analysis by LDI‐TOF MS is solving the protein aggregation issue.^[^ ^44^ ^]^ Because flying velocities of high‐mass ions are very slow in TOF experiments, a larger molecular size induced by aggregation can cause a low LDI efficiency and a decrease in sensitivity.^[^ ^44^, ^45^ ^]^ For quantitative and qualitative analysis of proteins, intact proteins have been cleaved enzymatically into small peptide fragments through a bottom‐up strategy; however, this approach almost inevitably increases labor and reduces accuracy.^[^ ^45^, ^46^ ^]^ Notably, in addition to acting as a metal ion stabilizer, CNC binds proteins to reduce protein aggregation at the gastric phase and reduces the mean particle size of proteins.^[^ ^47^ ^]^ The unique physicochemical properties of CNC suggest that AuNPs/CNC may improve the sensitivity and reproducibility of LDI‐TOF MS analysis by inhibiting protein aggregation, which we explored in this study. Finally, the AuNPs/CNC‐assisted LDI‐MS platform revealed differences in translational levels and was used to screen for potential protein markers of NSCLC (**Figure** **1**).

## 2 Results and Discussion

### 2.1 Aggregation of AuNPs is Suppressed in an AuNPs/CNC Solution

**Figure 2:**
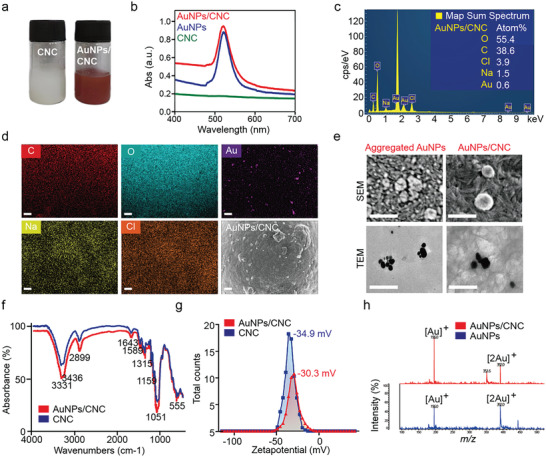
The construction and characterization of AuNPs/CNC. a) CNC and AuNPs/CNC dispersion solutions. b) UV‐vis spectrum of CNC, AuNPs and AuNPs/CNC solutions. c) EDX representative spectra of AuNPs/CNC. d) SEM image and energy mapping analysis of AuNPs/CNC. Scale bar, 500 nm. e) SEM and TEM images of AuNPs and AuNPs/CNC. Scale bar, 200 nm. f) FTIR spectra of CNC and AuNPs/CNC. g) Surface charge (zeta‐potential) of CNC and AuNPs/CNC. h) MS spectra of AuNPs/CNC and AuNPs at a low‐mass range.

AuNPs are extremely susceptible to aggregation, which reduces their ability to function fully. Thus, we synthesized CNC‐stabilized AuNPs to inhibit the formation of AuNP clusters. AuNPs were deposited onto CNC by reducing HAuCL_4_ with NaBH_4_. The AuNPs/CNC suspension exhibited a red color with an initial absorbance of 520 nm, known as the surface plasmon resonance peak. No absorption peak was observed in CNC solution. (**Figure** **2**a,b). Additionally, no AuNPs were detected by ultraviolet‐vis analysis in supernatants recovered after centrifugation. Figure 2c,d showed a representative energy dispersive X‐ray spectroscopy (EDX) spectrum and elemental mapping at the same sample as the scanning electron microscope (SEM) image itself. The characteristic Au peaks at 2.13 keV were found, while other peaks were attributed to CNC. Furthermore, the results presented in Figure 2e indicated that AuNPs were deposited evenly on the CNC surface, and the boundary between neighboring AuNPs was clearly apparent, whereas AuNPs underwent aggregation in the absence of CNC. The high number of carboxylic ions on CNC chains afford strong potential interactions with metal nanoparticles, and thus, AuNPs can be stable for several months in reaction systems.^[^ ^19^ ^]^ These results indicated that CNC facilitated high and stable AuNP loading onto CNC. In this reaction mixture, the HAuCL_4_ concentration and pH are pivotal in stabilizing AuNPs/CNC. As reported by Luong et al., no AuNPs were observed on the CNC surface when the HAuCL_4_/gold salt ratio was > 1.25 and the final solution pH was > 4.5. Hence, the optimal ratio of HAuCL_4_/gold salt in our work was determined to be 1:1, and the final pH was 3.0, which ensured the formation of stable AuNPs/CNC particles.^[^ ^48^ ^]^

Functional groups present in AuNPs/CNC were analyzed by Fourier transform infrared (FTIR) spectroscopy. FTIR peaks were observed at 3331 cm^–1^ (O–H stretching), 3436 cm^–1^ (β−1,4‐glucan chains of cellulose), 2899 cm^–1^ (C–H asymmetric stretching), 1643 cm^–1^ (O–H bending), 1589 cm^–1^ (C = O stretching vibration of protonated carboxyl groups), 1315 cm^–1^ (C–H_2_ wagging vibration), 1159 cm^–1^ (C–C ring stretching) and 1051 cm^–1^ (stretching vibration of C–O–C in the pyranose ring) (Figure 2f).^[^ ^49^, ^50^, ^51^ ^]^ Most functional groups were introduced through transient swelling of CNC, making the synthesized CNC more reactive for any downstream chemical modifications.^[^ ^52^ ^]^ Importantly, a significant reduction in the carboxyl groups (1589 cm^–1^) and hydroxyl groups was also observed in AuNPs/CNC, suggesting the positive conjugation of AuNPs onto CNC (Figure 2f). The zeta potential can be used to measure nanoparticle‐nanoparticle repulsion forces in colloidal suspensions and, thus, is used to predict colloid stability against particle aggregation.^[^ ^53^ ^]^ Conjugation of AuNPs was found to alter the zeta potential of CNC from –34.9 to –30.3 mV, indicating that conjugation of AuNPs onto CNC reduced the negative zeta potential (Figure 2g). Moreover, LDI‐TOF MS spectra of the AuNPs in the absence and presence of CNC are shown in Figure 2h and Figure S1 (Supporting Information). The Au ion and its dimers with m/z values of 196 and 392 were observed at the low‐mass range, indicating the presence of AuNPs (Figure 2h). As shown in Figure S1 (Supporting Information), AuNPs/CNC did not generate interference signals at the high‐mass range.

### 2.2 Protein Aggregation is Suppressed by Using AuNPs/CNC

**Figure 3:**
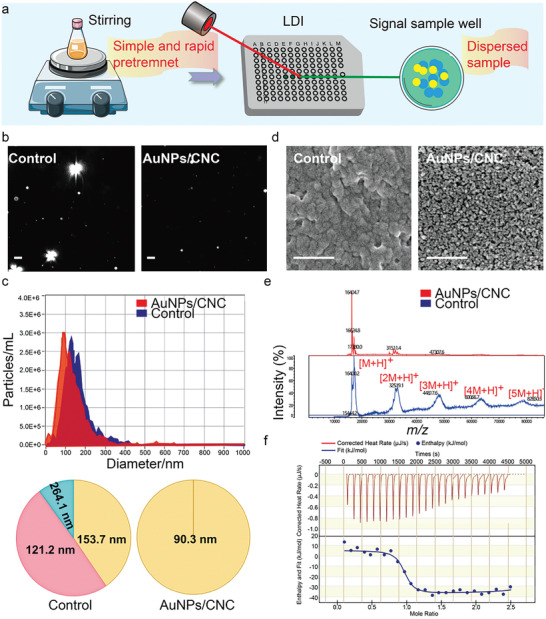
Protein aggregation is suppressed by AuNPs/CNC. a) Using AuNPs/CNC as the matrix material achieves simple and rapid preparation of dispersed samples for LDI‐TOF MS analysis. b) Laser scattering microscope images of 2 mg mL^–1^ Hb treated with or without 1 mg mL^–1^ AuNPs/CNC. Scale bar, 200 nm. c) Size distribution of 2 mg mL^–1^ Hb treated with or without 1 mg mL^–1^ AuNPs/CNC analyzed by NTA. d) SEM images of 2 mg mL^–1^ Hb treated with or without 1 mg mL^–1^ AuNPs/CNC. Scale bar, 200 nm. e) MS spectra of Hb with or without adding AuNPs/CNC. f) ITC analysis for the adsorption of Hb onto AuNPs/CNC.

Direct analysis of intact proteins is essential for accurate protein identification; however, protein aggregation induces a low desorption/ionization efficiency and a reduction in the sensitivity of LDI‐TOF MS analysis.^[^ ^45^ ^]^ Monomeric proteins in the sample associate with other proteins, forming oligomers as the initial aggregation species that eventually form higher multimer species.^[^ ^54^ ^]^ Related studies have reported electrostatic interactions between CNC and proteins, which shield aggregation “hot spots” and inhibit protein aggregation.^[^ ^55^ ^]^ For LDI‐TOF MS analysis of intact proteins, the mixture of AuNPs/CNC and protein sample was thoroughly stirred in a straightforward manner to inhibit protein aggregation (**Figure** **3**a). Hemoglobin (Hb) was used as the model protein to verify the feasibility of AuNPs/CNC for inhibiting protein aggregation. As shown in Figure 3b, Hb formed large aggregates, whereas the mean particle size of Hb was dramatically reduced after adding AuNPs/CNC. Nanoparticle tracking analysis (NTA) analysis indicated that the average diameter of Hb was reduced significantly after adding AuNPs/CNC to the sample when compared with the control group (90.3 nm vs 121.2–264.1 nm), demonstrating that protein aggregation was effectively reduced by AuNPs/CNC (Figure 3c; Table S1, Supporting Information). Also, results from SEM revealed that the presence of AuNP/CNC reduced the aggregate formation of Hb in the solutions when compared to control (Figure 3d). The reduction in protein size indicated that this approach should be beneficial for analyzing exosomal proteins from complicated clinical samples. In LDI‐TOF MS analysis, the existence of [nM+H]^+^ multiple peaks induces an ion inhibition effect and hampers the characterization of the major protein [M+H]^+^ peak. Next, the effects of AuNPs/CNC on intact protein analysis were investigated using LDI‐TOF MS. As shown in Figure 3e, prominent peaks indicative of aggregation were observed in the spectrum of Hb, including the dimer peak of *m/z* 31000, the trimer peak of *m/z* 46000 and the tetramer peak of *m/z* 61000. However, the signal intensity of the major protein [M+H]^+^ peak significantly increased and peaks representing aggregated species decreased when AuNPs/CNC was present (Figure 3e).

Isothermal titration calorimetry (ITC) can be used to analyze thermodynamic interactions between AuNPs and biological ligands such as nucleic acids and proteins. Based on ITC data, Guo et al. also reported a peptide sequence composed of seven residues interacting with CNC.^[^ ^56^ ^]^ We have shown that CNC inhibits protein aggregation in an aqueous medium. However, using CNC in biological systems requires further understanding of the interaction between CNC and complex biomolecules such as intact proteins. Furthermore, the binding of Hb to CNC with different degrees of substitution was analyzed through ITC. Figure 3f presents calorimetric traces obtained from adding 0.01 mM Hb to a suspension of 0.16 mM AuNPs/CNC and data fitting was based on a multiple‐sites model. The calorimetric traces yielded a negative Gibbs free energy, suggesting the process was entropically driven and endothermic binding between AuNPs/CNC and intact proteins.

### 2.3 Methodological Validation

**Figure 4:**
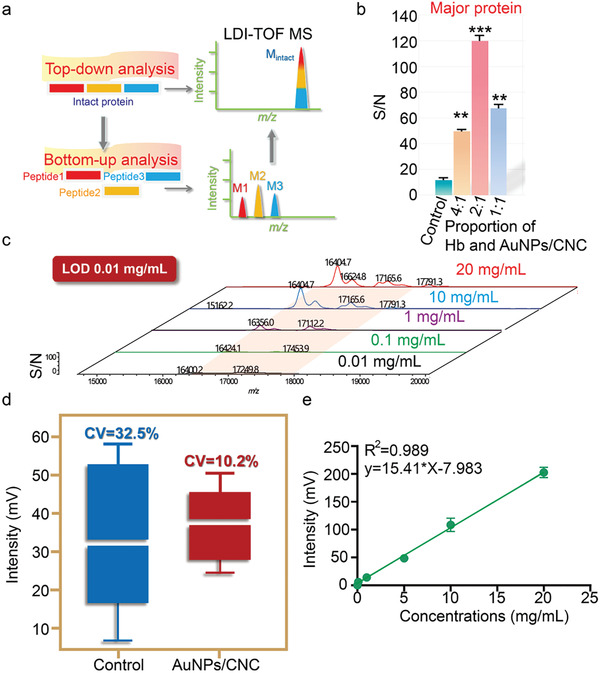
Methodological validation. a) Top‐down and bottom‐up strategies for protein analysis based LDI‐TOF MS. b) Statistical results of spectral S/N of different Hb and AuNPs/CNC proportions. The Hb concentrations were 1, 2 and 4 mg mL^–1^. ^**^ *p* < 0.01, ^***^ *p* < 0.001. c) MS of Hb at 0.01, 0.1, 1, 10 and 20 mg mL^–1^ after adding 10 mg mL^–1^ AuNPs/CNC. d) Repeatability analysis of Hb ion signals from 15 sample wells in the presence or absence of AuNPs/CNC. e) Linear relationship between the signal intensity and the concentrations of Hb after adding AuNPs/CNC.

For quantitative and qualitative analysis of intact proteins (the top‐down strategy), the sensitivity and reproducibility of LDI‐TOF MS needed to be further improved. In the conventional strategy for protein analysis, intact proteins are cleaved enzymatically into small peptides through a bottom‐up assay, which increases labor and reduces accuracy (**Figure** **4**a). By constructing the AuNPs/CNC matrix material, we hoped to achieve direct analysis of intact proteins by LDI‐TOF MS. The signal‐to‐noise ratio (S/N) of the Hb spectrum increased by two orders of magnitude using AuNPs/CNC compared with the control (Figure 4b). The limit of detection (LOD) to clearly distinguish any peak as a signal from the noise was defined as S/N > 3. The S/N of the Hb standard was in line with the concentrations (ranging from 0.01 to 20 mg mL^–1^), and the LOD was ≈0.01 mg mL^–1^ after adding AuNPs/CNC as the matrix (Figure 4c), indicating much higher sensitivity for intact protein analysis. In general, quantification using LDI‐TOF MS remains challenging because of ion signal inhomogeneities. Unfortunately, protein aggregation may be observed at all stages of sample preparation and analysis, which further increases the variability of the analysis results. Compared with the control group, the use of AuNPs/CNC not only enhanced the ion intensity but also contributed to good reproducibility in the LDI‐TOF MS analysis, in which the CV of ion signals with or without AuNPs/CNC was 10.2% and 32.5%, respectively (Figure 4d). Encouraged by the improvement in signal sensitivity and reproducibility, an excellent linear relationship was obtained over the range of 0.1–20 mg mL^–1^ after adding AuNPs/CNC to the sample, suggesting that AuNPs/CNC can be used for quantitative analysis of intact proteins through LDI‐TOF MS (Figure 4e).

### 2.4 Analysis of Serum Exosomes from NSCLC Patients

**Figure 5:**
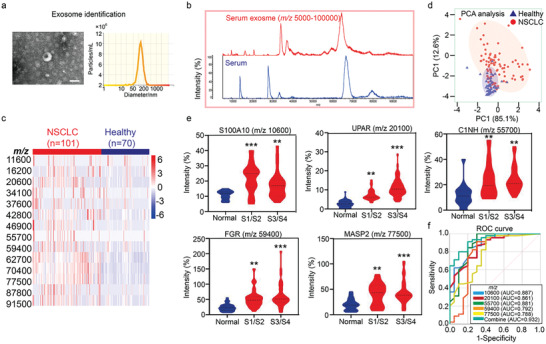
Analysis of serum exosome proteins based AuNPs/CNC assisted LDI‐MS. a) TEM images of exosomes isolated from human serum. Scale bar, 200 nm (left), and concentrations and NTA of the size distribution of exosomes isolated from human serum (right). b) Comparison of the spectra of serum exosomes and unprocessed serum using AuNPs/CNC assisted LDI‐TOF MS. c) Heat map of the distribution of the 14 features in 171 serum exosome samples (101 NSCLC and 70 controls). d) PCA clustering diagram of the 171 serum exosome samples (101 NSCLC and 70 controls). e) Violin plots showing the relationships between S100A10, UPAR, C1NH, FGR and MASP2 ion intensities and pathological stages. ^**^ *p* < 0.01, ^***^ *p* < 0.001. f) ROC curve analysis of the five exosome‐related peaks.

Serum samples from 70 healthy individuals and 101 NSCLC patients were included to assess the potential and clinical value of AuNPs/CNC for LDI‐TOF MS analysis of exosomal proteins. The isolated exosomes had a typical saucer‐like membrane structure and a single peak with a mean size of 120 nm (**Figure** **5**a). Exosomal lysates were then directly analyzed by LDI‐TOF MS at the high‐mass range (*m/z* 5000–100000). As shown in Figure 5b, significant differences were observed between protein fingerprints of serum and serum exosomes, indicating the validity of serum exosome isolation and analysis. Because of the individual differences, the obtained signal peak number was 20 in each exosomal sample, from which 14 reliable peaks with ion intensity > 10 were chosen for subsequent analysis. Heat maps of the 14 features on the 101 NSCLC and 70 control samples are presented in Figure 5c, and distinct differential expressions between the two groups were observed. Thus, the specific fingerprints of exosome proteins provide a potential approach to diagnosing NSCLC. High‐dimensional protein MS data were performed using principal component analysis (PCA), and the first (83.2%) and the second (13.4%) principal components were chosen for visualization. Clear separation of NSCLC and healthy controls was observed in the PCA model, showing heterogeneity between the two groups (Figure 5d). An ideal tumor marker is associated with clinical characteristics. Moreover, we found that the signal intensity of five exosome proteins was upregulated and remained significantly associated with the T stage of NSCLC patients (Figure 5e; Figures S2–S4, Supporting Information). According to the bottom‐up proteomic analysis, five typical peaks were identified as *m/z* 10600 S100 calcium‐binding protein A10 (S100A10) (Uniprot ID: P60903), *m/z* 20100 urokinase plasminogen activator surface receptor (UPAR) (Uniprot ID: Q03405), *m/z* 55700 plasma protease C1 inhibitor (C1NH) (Uniprot ID: P05155), *m/z* 59400 Tyrosine‐protein kinase Fgr (FGR) (Uniprot ID: P09769) and *m/z* 77500 mannose‐binding lectin associated serine protease 2 (MASP2) (Uniprot ID: O00187) (Table S2, Supporting Information). Finally, the receiver operating characteristic (ROC) curve was used to evaluate the diagnostic value of the five characteristic exosomal proteins in NSCLC. As expected, the ROC analysis revealed that the five protein peaks can be used to predict NSCLC: the combined multi‐index diagnosis area under the curve was 0.932. (Figure 5f). S100A10 regulates protein phosphorylation and cell signal transduction. S100A10 has been reported to be overexpressed in lung cancer tissues and promotes the malignant phenotype by inducing matrix metalloproteinase (MMP) 2 and MMP9, which can promote extracellular matrix degradation and cell migration.^[^ ^57^ ^]^ UPAR is a glycated phosphatidylinositol‐anchored protein that mediates the degradation of the extracellular matrix and basement membrane.^[^ ^42^ ^]^ UPAR interacts with different transmembrane proteins to activate intracellular signals mediated by mitogen‐activated protein kinase and further promotes the initiation and progression of many types of human cancer.^[^ ^58^ ^]^ Moreover, high levels of UPAR in peripheral blood and tumor tissue are closely associated with the poor prognosis of patients.^[^ ^59^ ^]^ MASP2, as a member of the serine proteases family, is a key regulator in the complement‐activated lectin pathway. Previous studies have indicated that MASP2 gene mutations and changes in the serum MASP2 content are correlated with the prognosis of patients with lung, colorectal, liver, and esophageal cancer.^[^ ^60^, ^61^ ^]^ C1NH regulates the activation of the C1 complex, which is pivotal in regulating important physiological pathways, including complement activation, venous thrombosis, and kinin generation. Additionally, accumulating evidence indicates the presence of complement components and some regulatory molecules in most carcinoma cells. Hence, analysis of C1NH levels has potential value for the diagnosis and prognosis of related human diseases.^[^ ^62^ ^]^ FGR is a nonreceptor tyrosine‐protein kinase with broader functions for lung disease. FGR is able to phosphorylate/activate phosphatidylinositol 3‐kinase receptor 1, and overexpression of FGR in NSCLC, especially in adenocarcinomas, may play a pivotal role in the NSCLC progression.^[^ ^63^ ^]^ In our study, these exosomal proteins were identified as potential prognostic markers for NSCLC patients through LDI‐TOF MS analysis, which provides a solid foundation for developing a platform that uses exosomes as a targeted protein phenotypic tool in clinical studies.

### 2.5 Construction of the Nomogram for NSCLC Diagnosis

**Figure 6:**
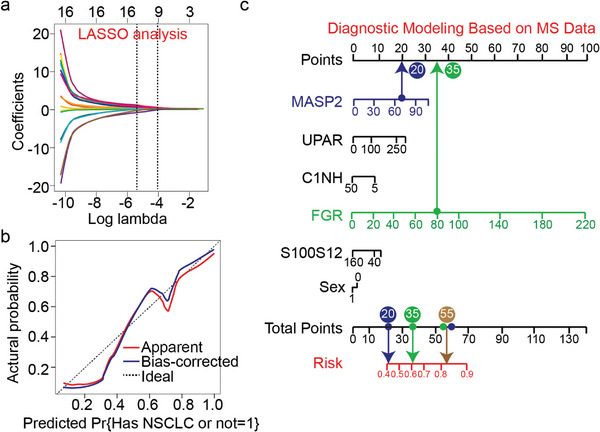
Construction of the nomomodel for NSCLC diagnosis. a) A coefficient profile plot was constructed against the log (lambda) sequence. b) Calibration curve analysis of the nomogram. c) Nomogram predicting the diagnostic value of serum exosome‐related peaks for NSCLC.

Least absolute shrinkage and selection operator (LASSO) regression analysis was performed to select predictive variables from the clinical characteristics (sex and age) and the 14 proteins identified in serum exosomes (Table S2, Supporting Information). Six of the 16 variables were included in the predictive model, namely sex and ion intensity of S100A10, UPAR, C1NH, FGR, and MASP2, which had non‐zero coefficients (**Figure** **6**a,b). Moreover, the nomogram was used to quantitatively predict the risk probability of NSCLC based on the LASSO regression model (Figure 6c). For example, using the nomogram model, a patient with a MASP2 ion intensity of 20 and a FGR ion intensity of 35 had an estimated 55% risk of NSCLC (Figure 6c). In summary, these results confirmed the potential utility of AuNPs/CNC as a matrix for LDI‐TOF MS analysis of intact proteins from NSCLC exosomes.

## 3 Conclusion

AuNPs/CNC with a “dual dispersion” effect served as an excellent matrix for intact protein analysis using LDI‐TOF MS with high sensitivity and excellent reproducibility. Using CNC effectively dispersed and stabilized AuNPs in the reaction system. The signal intensity and point‐to‐point reproducibility of analytes were improved by inhibiting the production of Au cluster ions. An important innovative application of AuNPs/CNC was the analysis of intact protein using the LDI‐TOF MS platform. Interactions between AuNPs/CNC and proteins reduced protein aggregation effectively in the sample, which increased the molecular ion [M+H]^+^ signal and decreased aggregated ion [nM+H]^+^ signals in LDI‐TOF MS analysis. Moreover, the homogeneity of analytes was improved significantly using AuNPs/CNC, which contributed to excellent point‐to‐point reproducibility for LDI‐TOF MS analysis. The AuNPs/CNC nanocomposite was successfully applied to analyze intact proteins extracted from serum exosomes of NSCLC patients. LDI‐TOF MS analysis afforded an exosome protein fingerprint that effectively distinguished between healthy individuals and NSCLC patients. The diagnostic model constructed using the MS data identified exosomal proteins S100A10, UPAR, C1NH, FGR, and MASP2 as excellent predictive biomarkers of NSCLC risk.

## 4 Experimental Section

### Samples Collection and Preparation

All serum samples were collected from the Department of Clinical Laboratory Medicine, Shanghai Chest Hospital. The study was conducted in accordance with the Declaration of Helsinki, and the protocol was approved by the Medical Ethics Committee of Shanghai Chest Hospital (No. KS(Y)23 029). The 171 serum samples were divided into the NSCLC group, which included 101 NSCLC serum samples, and the control group, which included 70 serum samples from healthy individuals. Serum samples were transported in holding tanks filled with dry ice and stored at –80 °C before analysis.

### Preparation of the AuNPs/CNC Conjugate

AuNPs/CNC were prepared according to a previous method.^[^ ^36^ ^]^ Initially, 10 mM HAuCl_4_ (2 mL) and 2 mM α‐cyclodextrin (2 mL) were added to 4 mL CNC (20 mg mL^–1^). Twenty microliters of 0.1 M NaBH_4_ were added, and the solution became reddish immediately. The AuNPs/CNC were washed and centrifuged at 10,000 *g* three times after stabilizing for 24 h at room temperature. The characterization was determined by UV‐vis and FTIR spectroscopies and confirmed by SEM and transmission electron microscopy (TEM). Elemental analysis of maps of wereas revealed by SEM was performed by the SEM/EDX system (Oxford Instruments, UK). The surface charge of AuNPs/CNC was measured by electrophoretic light scattering with a NanoBrook 90Plus Zeta instrument (Brookhaven, USA). The protein and AuNPs/CNC mixture with different concentration ratios were stirred thoroughly at 1000 rpm, and MS analysis was performed rapidly thereafter. The size of protein granules was detected by NTA and a laser scattering microscope.

### Exosomal Isolation and Identification

Serum exosomes were isolated according to the commercial kit (Umibio, China). In brief, exosomes were isolated from 500 µL serum and resuspended in 200 µL phosphate‐buffered saline. The exosomal solution was then treated with a cell lysate (Solarbio, China) containing 1% phenylmethylsulfonyl fluoride (5:2, v/v) at 4 °C for 40 min. After centrifugation at 12,000 *g* for 20 min at 4 °C, the supernatants were collected as exosome protein samples. TEM was performed to analyze the morphology, and NTA was used to detect the size and concentrations.

### LDI‐TOF MS Analysis

Before LDI‐TOF MS analysis, 1 µL of a mixture containing analytes and matrix solution was placed onto the sample plate and dried at room temperature. Synaptic acid was used as the matrix in a non‐nanoparticle control group. Spectra were collected using a Shimadzu Biotech Launchpad MALDI‐TOF MS (Shimadzu, Japan) in the linear mode. Ionization was achieved by a 337‐nm N_2_ laser, and spectra were calibrated using external standards.

### ITC Analysis

Calorimetric experiments were performed using a NANO Isothermal Titration Calorimeter (TA Instruments, USA). The AuNPs/CNC concentration was 0.16 mM, and the protein standard concentration was 0.01 mM. The initial volumes of the AuNP/CNC and Hb samples were 50 and 350 µL, respectively. Experiments used the following parameters: 25 drops (i.e., data points), 2 µL per drop, a 180‐s titer interval, an agitation speed of 350 rpm, and 25 °C. Nonlinear regression was used along with model equations to determine the best‐fitting parameter values.

### Statistical Analysis

Data were expressed as mean ± SD. Differences between the AuNPs/CNC and control groups were analyzed using the student's *t*‐test. Differences between the AuNPs/CNC and control groups were analyzed using the student's t‐test (SPSS 21.0). Comparisons between two populations were performed using two independent sample t‐tests (SPSS 21.0). A *p* < 0.05 was considered statistically significant. PCA and ROC curve analyses were performed based on LDI‐TOF MS data with SPSS 21.0. LASSO regression analysis was performed to fit the data, and the best lambda value was selected. Logistic regression was then performed using the R package (Logreg 6.2.0) to construct a predictive model for NSCLC. All included factors were applied to develop the nomogram prediction model.

## Conflict of Interest

The authors declare no conflict of interest.

## Supporting information

Supporting Information

## Data Availability

The data that support the findings of this study are available from the corresponding author upon reasonable request.
