# Supplementary material for: AuNPs/CNC Nanocomposite with A “Dual Dispersion” Effect for LDI‐TOF MS Analysis of Intact Proteins in NSCLC Serum Exosomes
Source: Adv Sci (Weinh). 2024 Jan 15;11(12):2307360. doi: 10.1002/advs.202307360 (PMC10966532; doi:10.1002/advs.202307360)
Supplement: Supplementary file 1 — Supporting Information [file ADVS-11-2307360-s001.pdf]

ADVANCED SCIENCE
Open Access

Supporting Information

for *Adv. Sci.*, DOI 10.1002/advs.202307360

AuNPs/CNC Nanocomposite with A "Dual Dispersion" Effect for LDI-TOF MS Analysis of Intact Proteins in NSCLC Serum Exosomes

Liang Shan, Yongxia Qiao, Lifang Ma, Xiao Zhang, Changqiang Chen, Xin Xu, Dan Li, Shiyu Qiu, Xiangfei Xue, Yongchun Yu, Yinlong Guo*, Kun Qian* and Jiayi Wang**

Supporting Information

AuNPs/CNC Nanocomposite with a "Dual Dispersion" Effect for LDI-TOF MS Analysis of Intact Proteins in NSCLC Serum Exosomes

Liang Shan#, Yongxia,Qiao#, Lifang Ma#, Xiao Zhang, Changqiang Chen, Xin Xu, Dan Li, Shiyu Qiu, Xiangfei Xue, Yongchun Yu*, Yinlong Guo*, Kun Qian*, Jiayi Wang***Table S1.** NTA peak analysis of Hb with or without AuNPs/CNC.

Group	Diameter (nm)	Particles/mL	Full width at half maxima	Percentage
Control	121.2	2.8E+6	52.8	49.2
	153.7	2.4E+6	38.0	40.9
	264.1	4.1E+5	37.2	9.9
AuNPs/CNC	90.3	3.1E+6	98.8	100

Table S2. The 14 specific proteins identified by shotgun proteomics.

m/z	Uniprot ID	Protein name	Sequence coverage (%)	Intensity (Quantified)
10600	P60903	S100A10	43	392930
16200	P69905	Hb α	15.6	66115
20100	Q03405	UPAR	20.6	60628
34100	P25311	Zinc α glycoprotein	15.1	393610
37600	P02042	Hb delta	46.9	70479
42800	P68133	Skeletal muscle α	19.1	217310

46900	Q9H3U5	Major facilitator superfamily domain-containing protein 1	3	556070
55700	P05155	C1NH	35.2	953670
59400	P09769	FGR	46.7	27408000
62700	Q14520	Hyaluronan binding protein 2	16.4	1152700
70400	P00734	Prothrombin	44.9	25907000
77500	O00187	MASP2	33.2	6478000
87800	P06396	Gelsolin	41.7	33144000
91500	P80108	Phosphatidylinositol-glycan-specific phospholipase D	15.6	66115

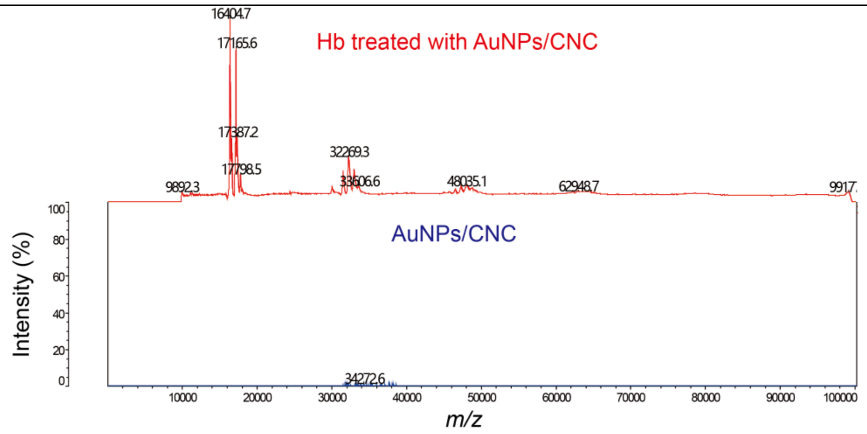

Figure S1. MS spectra of AuNPs/CNC and Hb treated with AuNPs/CNC at a high-mass range.

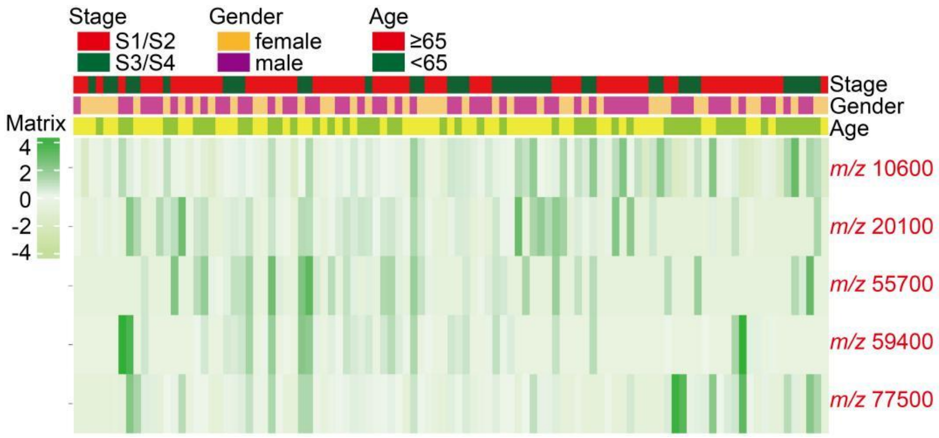

Figure S2. Clinical heatmap of the distribution of the five features in 101 NSCLC patients.

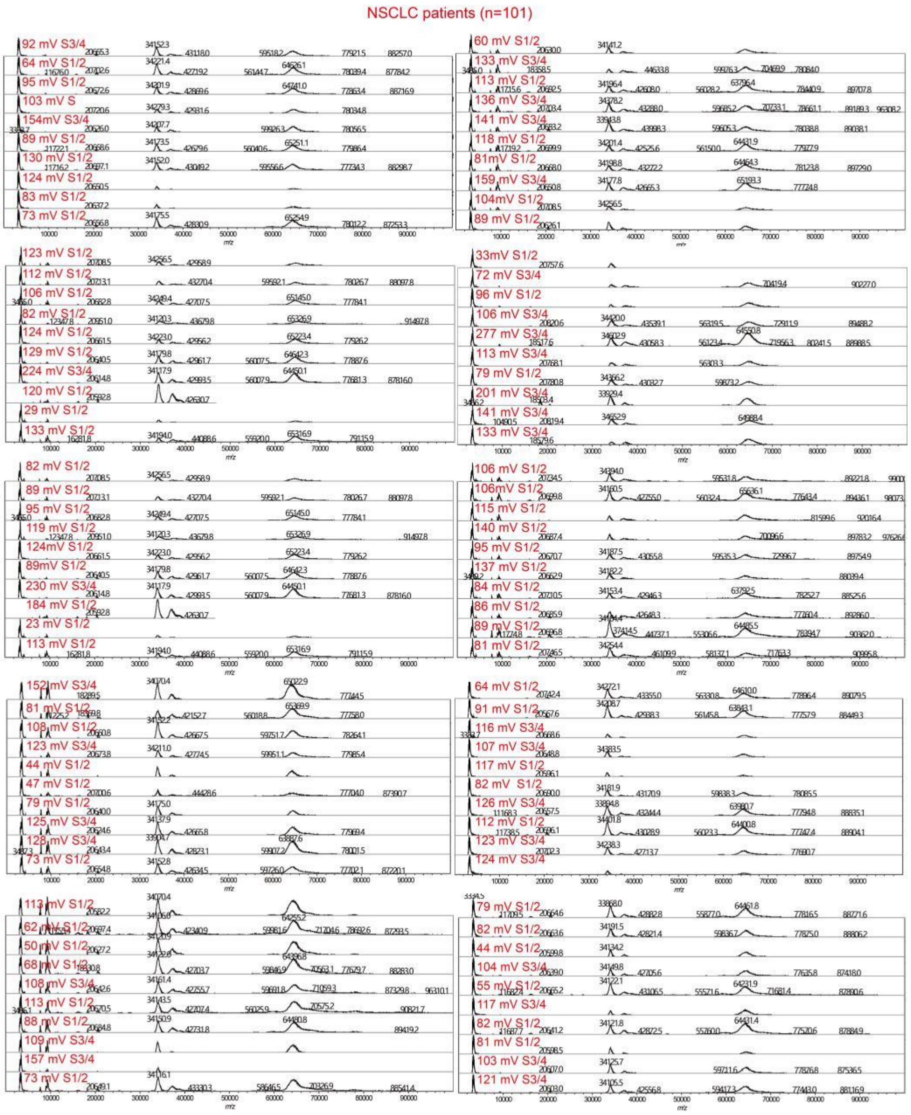

Figure S3. Intact protein spectra of serum exosomes from the 101 patients with NSCLC. Ionic signal intensity (mV) and corresponding tumor stages (S1/2 or S3/4) are shown in the spectra.

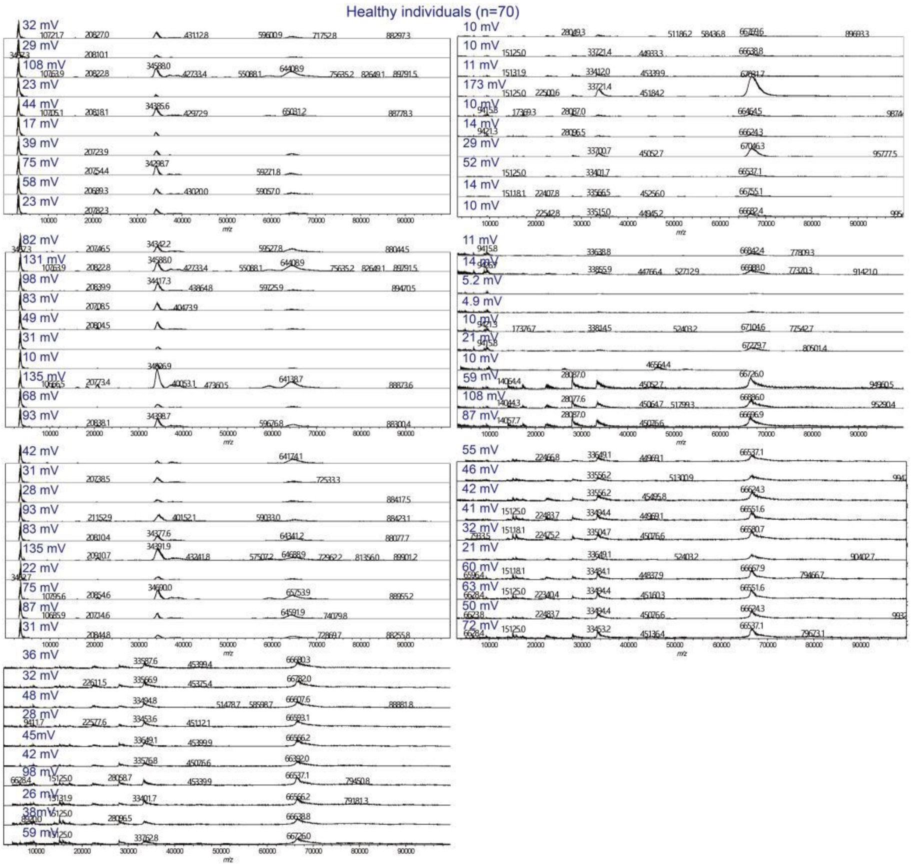

Figure S4. The intact protein spectra of serum exosome from the 70 healthy individuals. Ionic signal intensity (mV) are shown in the spectra.
